# Imported Measles and Implications for Its Elimination in Taiwan

**DOI:** 10.3201/eid1708.100800

**Published:** 2011-08

**Authors:** Wen-Yueh Cheng, Chen-Fu Yang, Yu-Ting Hou, Shih-Chuan Wang, Hsiu-Li Chang, Hsien-Ya Chiu, En-Tzu Wang, Ho-Sheng Wu

**Affiliations:** Author affiliation: Centers for Disease Control, Taipei, Taiwan

**Keywords:** viruses, measles, Taiwan, nosocomial, measles outbreak, importation, dispatch

## Abstract

During November 2008–May 2009, an outbreak of 53 measles cases occurred in Taiwan. Of these, 3 cases were sporadic, and the other 50 cases could be grouped into 8 clusters by genetic analysis. We determined 7 H1 genotypes linked to importation and 1 G3 genotype linked to an untraceable source.

The availability of a measles vaccine in the 1960s led to substantial reductions in the incidence of measles globally. Along with improvements in living conditions, nutrition, and the availability of antimicrobial drugs for secondary bacterial infections, the mortality rate was greatly reduced (1). A live-attenuated measles vaccine was introduced to Taiwan in 1968, and a routine vaccination policy was established in 1978 to provide measles vaccine to infants at 9 and 15 months of age. During 1992–1994 and 2001–2004, two catch-up campaigns were implemented, targeting birth cohorts September 1976 to September 1990 and September 1990 to September 1994, respectively. Starting in 2006, the measles, mumps, and rubella (MMR) vaccine targeted infants 12–15 months old and 6-year-old children. The coverage rates for the first dose of measles vaccine and the second dose of MMR were 91% and 95%, respectively, since 1996. In recent decades in Taiwan, 4 major measles outbreaks have occurred, with 2,219 (in 1985), 1,386 (1988), 1,060 (1989), and 303 (1992) reported cases. Beginning in 1993, the annual number of reported measles cases was <100; by the end of 2007, the annual number of confirmed measles cases was <10 (2). The ability to actively control measles has increased in Taiwan since 1991 because of a combined plan to eliminate polio, measles, congenital rubella syndrome and neonatal tetanus.

The key to controlling measles is achieving and sustaining high levels of vaccination coverage. Finding a way to prevent the transmission of measles is an important topic for any country in the elimination phase. Here, we report several measles outbreaks that occurred in Taiwan during 2008 and 2009.

## The Study

In total, 140 suspected measles cases were reported to the Taiwan Centers for Disease Control from November 2008 to May 2009, and 53 were confirmed (Table A1). The criteria for confirming cases included laboratory diagnosis and establishing an epidemiologic link to the laboratory-diagnosed case. The criteria for the laboratory diagnosis included the presence of measles immunoglobulin (Ig) M, isolation of measles virus, or identification of measles virus by reverse transcription PCR (2,3).

**Figure 1:**
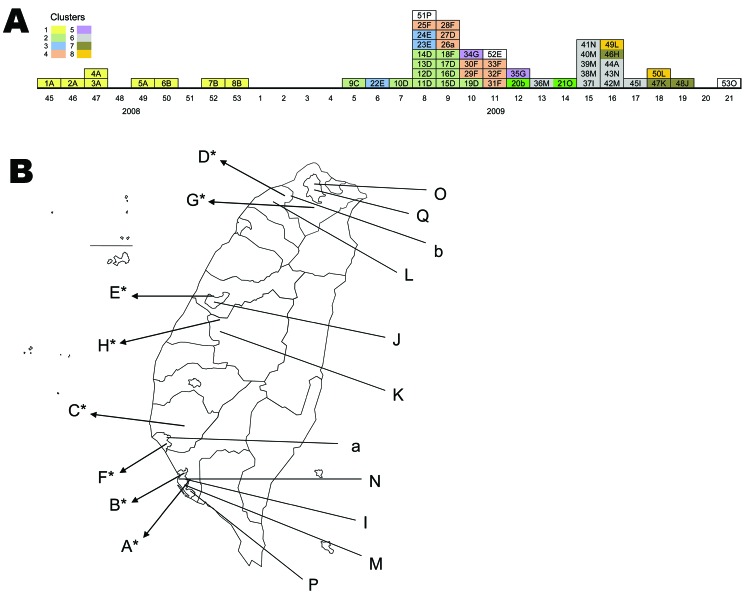
A) Timetable of 53 measles cases reported in Taiwan from week 45 of 2008 to week 21 of 2009. Each cell in the timetable represents 1 case; each case is identified with an identification number followed by a letter that represents the hospital (uppercase) or clinic (lowercase) that reported the case. Cases 1–8, 9–21, 22–24, 25–33, 34–35, 36–45, 46–48, and 49–50 belong to clusters 1 through 8, respectively, and cells within a cluster are the same color. Cases 51–53 were from 3 sporadic cases and are not colored. The case numbers peaked during the eighth week of 2009, with 8 cases distributed through northern (hospital D), central (hospital E), and southern (hospitals F and P) Taiwan; these cases originated from 4 importation chains. B) Locations of hospitals involved in measles outbreaks. *Hospital was involved in nosocomial transmission.

A timetable for occurrence of the 53 measles cases was constructed (Figure 1, panel A) on the basis of the week of onset of rash for each patient. The locations of the hospitals that reported these cases are shown in Figure 1, panel B.

**Figure 2:**
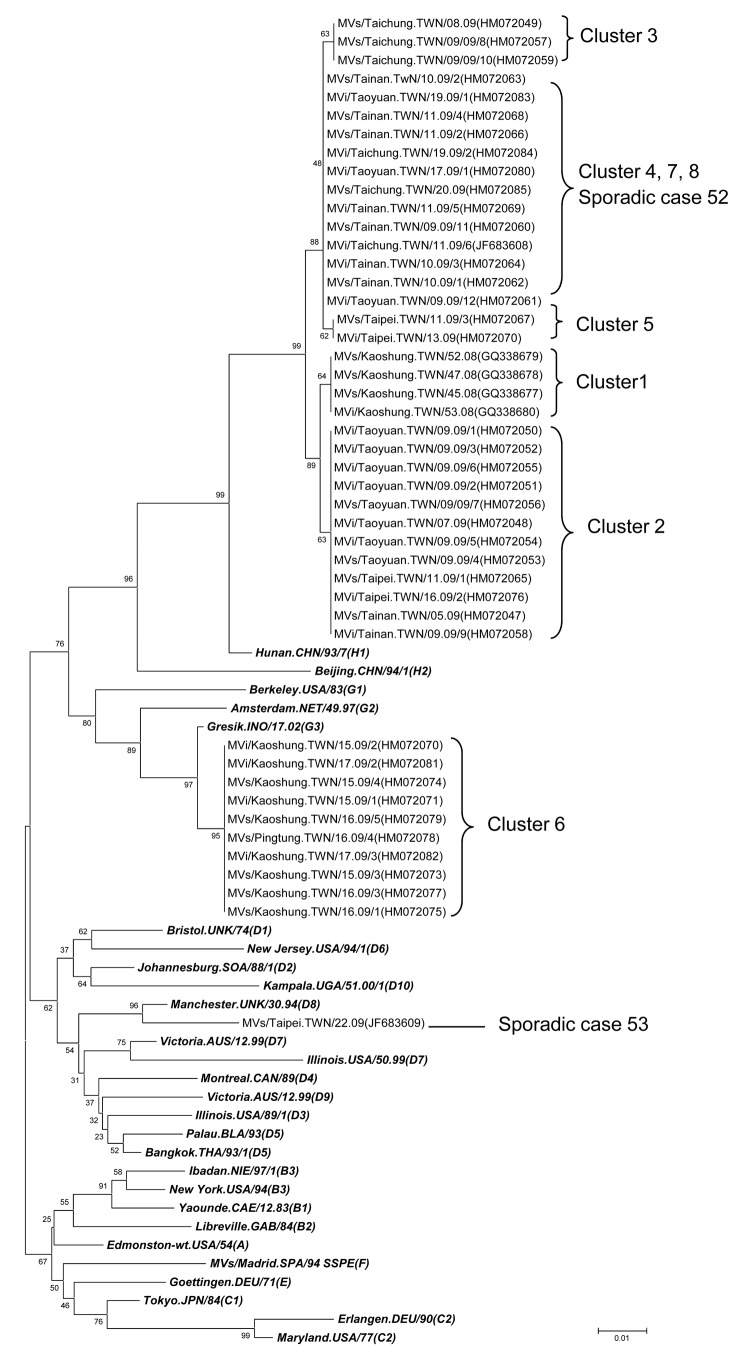
Phylogenetic analyses of the 456 carboxyl-terminal nucleotides of the N gene sequences of isolates obtained from 45 measles case-patients from November 2008 through May 2009, Taiwan. The respective accession number for each sequence is shown in parentheses following the strain name. ***Boldface italics*** indicate World Health Organization reference strains. The unrooted neighbor-joining consensus tree was generated by bootstrap analysis of 1,000 replicates by using MEGA4 software (www.megasoftware.net). Scale bar indicates nucleotide substitutions per site.

Cluster 1 involved nosocomial infections in hospital A (case-patients 1–5) and hospital B (case-patients 6–8); the transmission between case-patient 5 and case-patient 6 was through household contact. The index case-patient (case 7) was discovered later. Cluster 2 (case-patients 9–21) caused nosocomial infections in hospitals C (case-patients 9–10) and D (case-patients 10–17 and 19). Case-patient 18 was reported by hospital F, case-patient 20 from clinic b and case-patient 21 from hospital O. With the exception of case-patient 20, who attended kindergarten with case-patient 19, there was no exposure linkage between case-patients 18 and 21; the phylogenic data (Figure 2) did, however, indicate the patients were infected with related strains. Cluster 3 involved case-patients 22–24; the index case-patient was the source of transmission and caused the nosocomial infection in hospital E. Cluster 4 (case-patients 25–33) caused nosocomial infections in hospital F (case-patients 25 and 27–33), and case-patients 25 and 26 attended the same school. Cluster 5 (case-patients 34–35) was a nosocomial infection in hospital G. Cluster 6 involved 10 cases: the index case-patient, case 37, was reported by hospital I. Case-patients 36–43 were from the same military base, and case-patients 37, 44, and 45 were relatives. Cluster 7 caused nosocomial infections in hospital H (case-patients 46 and 47) as well as household transmissions (case-patients 47 and 48). Cluster 8 (case-patients 49 and 50) was transmitted by contact at a dormitory. Case-patient 51 had a sporadic case from an unidentified source; case-patients 52 and 53 had sporadic cases linked to importation from Vietnam and India, respectively.

Of these 53 patients with confirmed measles, 36 (67.9%) were male. There were 15 children (28.3%) <12 months of age and 19 children (35.8%) from 12 months to 6 years of age. Adults accounted for 19 cases (35.8%). Only 1 patient (1.88%) had received 2 doses of a measles-containing vaccine; 4 patients (7.5%) had received 1 dose; 7 patients (13.2%) recalled having received vaccines; 9 adults (17.0%) did not know their vaccination status; and the remaining 31 patients (58.4%) had not received any vaccines. Among the 6 nosocomial measles outbreaks, 2 infection sources (clusters 2 and 5) were children <12 months old and therefore not eligible for the first dose of the MMR vaccine, 4 clusters (clusters 1, 3, 4, and 7) were caused by children between 13–17 months old who were not vaccinated, and 1 cluster (cluster 8) was caused by an adult from Vietnam with an uncertain vaccination history. The outbreak on the military base was caused by an adult who was assumed to have been vaccinated.

Phylogenic analysis determined that the measles virus genotypes from these 8 outbreaks fell into 6 clades (Figure 2). Although the source was untraceable, the measles virus sequences from cluster 6 belonged to genotype G3; the sequence was 100% identical to a G3 strain (MVi/H Kajang.MYS/11.09) isolated from Malaysia (T. Tran, pers. comm.). The other sequences all belonged to genotype H1 and were grouped into 5 lineages. Clusters 1, 2, and 3 were linked to importation from the People’s Republic of China and were grouped into different lineages. Clusters 4, 5, 7, and 8, which originated in Vietnam, were divided into 2 lineages, with clusters 4, 7, and 8 in the same lineage and cluster 5 in another lineage.

## Conclusions

Among these 53 confirmed cases, 26 (49%) patients contracted measles from hospital exposure, and 3 patients (5.7%) were hospital staff. Vaccination policies for workers in health care institutes should be strengthened because these workers have the highest potential risk of contracting the disease (4,5). Many other febrile exanthematic diseases caused by pathogens other than measles virus, such as enterovirus, rubella virus, parvovirus B19, human herpesvirus-6, or Kawasaki syndrome, could have symptoms similar to measles and be misdiagnosed (6–8). It is therefore vital to ensure that clinicians are fully aware of the disease and can recognize it in a timely manner.

After import-associated measles outbreaks were recognized, the MMR vaccination was recommended for persons planning to travel to measles-endemic countries. Successful immunization policies that include routinely vaccinating children with 2 doses of a measles-containing vaccine greatly reduce disease contraction among preschool and primary school children (9–11). Because measles outbreaks may occur in areas with high vaccine coverage (12–14), the higher proportion of measles cases in adults, especially in young adults, should be addressed further and evaluated with regard to the possible need for additional immunizations.

The major threats to measles control in Taiwan are similar to those faced by other countries in the elimination phase; the key questions are 1) how to detect the index case in real time, and 2) how to prevent import-associated transmission. Among these continuous outbreaks, 6 chains originated from children <18 months of age without adequate immunization who had recently traveled to measles-endemic countries. Clinicians being alert for patients being brought for treatment with a fever and rash at pediatric clinics will greatly improve the sensitivity of measles surveillance systems and prevent further nosocomial transmission. Special emphasis should be paid to pediatric patients without vaccination records and with a history of travel abroad. Of course, the ultimate goal of eliminating measles requires an agreement from all countries to contain the disease in their own territories.

## Appendix

**Table A1:** Characteristics of 53 measles case-patients in outbreaks in Taiwan, November 2008–May 2009*

Classification (year) and case no.	Patient sex	Patient age, y/mo	Date of rash onset	Date of sampling	Vaccination‡	Genotype	Strain§	Immunoglobulin¶	
								M	G
Cluster 1 (2008)									
1	F	01/05	Nov 4	Nov 6	No	H1	MVs/Kaohsiung.TWN/45.08	ND	ND
2	M	00/11	Nov 15	Jan 22†	No	–	–	E	P
3	F	04/05	Nov 17	Nov 21	No	H1	MVs/Kaohsiung.TWN/47.08	P	P
4	F	00/09	Nov 20	Jan 22†	No	–	–	E	P
5	M	02/05	Dec 2	Jan 17†	No	–	–	P	P
6	M	02/05	Dec 13	Dec 16	No	–	–	P	P
** 7**	M	00/09	Dec 23	Dec 26	No	H1	MVs/Kaohsiung.TWN/52.08	P	N
8	F	39/11	Dec 29	Dec 31	No	H1	MVi/Kaohsiung.TWN/53.08	P	P
Cluster 2 (2009)									
9	F	00/11	Jan 27	Jan 28	No	–	MVs/Tainan.TWN/05.09	P	P
** 10**	M	05/05	Feb 9	Feb 11	Yes	H1	MVi/Taoyuan.TWN/07.09	P	N
11	F	02/01	Feb 17	Feb 22	No	H1	MVi/Taoyuan.TWN/09.09/2	P	N
12	M	00/09	Feb 18	Feb 22	No	H1	MVi/Taoyuan.TWN/09.09/3	P	N
13	M	01/06	Feb 21	Feb 22	No	H1	MVs/Taoyuan.TWN/09.09/4	P	N
14	M	00/10	Feb 21	Feb 22	No	H1	MVi/Taoyuan.TWN/09.09/5	P	N
15	M	00/10	Feb 22	Feb 22	No	H1	MVi/Taoyuan.TWN/09.09/1	P	N
16	M	00/10	Feb 22	Feb 23	No	H1	MVi/Taoyuan.TWN/09.09/6	N	N
				Mar 3				P	P
17	F	02/05	Feb 24	Feb 24	No	H1	MVs/Taoyuan.TWN/09.09/7	P	N
18	F	39/10	Feb 23	Feb 26	Unknown	H1	MVi/Tainan.TWN/09.09/9	P	E
19	M	05/05	Mar 4	Mar 10	**Yes**	H1	MVs/Taipei.TWN/11.09/1	P	E
20	F	06/03	Mar 15	Mar 18	Yes (2 doses)	–	–	P	P
21	M	03/02	Apr 2	Apr 14	No	H1	MVi/Taipei.TWN/16.09/2	P	N
Cluster 3 (2009)									
** 22**	M	01/04	Feb 6	Feb 20	**Yes**	H1	MVs/Taichung.TWN/08.09	P	N
23	F	01/04	Feb 20	Feb 25	No	H1	MVs/Taichung.TWN/09.09/8	P	N
24	M	00/09	Feb 17	Feb 26	No	H1	MVs/Taichung.TWN/09.09/10	P	P
Cluster 4 (2009)									
25	F	01/01	Feb 15	Mar 3	No	–	–	P	P
** 26**	M	02/03	Feb 17	Feb 26	No	H1	MVs/Tainan.TWN/09.09/11	P	N
27	M	31/11	Feb 24	Feb 28	*Yes*	H1	MVi/Taoyuan.TWN/09.09/12	P	N
28	M	00/08	Feb 27	Mar 2	No	H1	MVs/Tainan.TWN/10.09/2	P	N
29	M	29/06	Feb 28	Mar 1	Yes	H1	MVs/Tainan.TWN/10.09/1	N	P
				Mar 16				P	P
30	F	00/11	Mar 1	Mar 4	No	H1	MVi/Tainan.TWN/10.09/3	P	N
31	F	36/04	Mar 8	Mar 10	Unknown	H1	MVs/Tainan.TWN/11.09/2	P	P
32	M	23/07	Mar 13	Mar 13	**Yes**	H1	MVs/Tainan.TWN/11.09/4	N	P
33	M	26/06	Mar 13	Mar 13	Unknown	H1	MVi/Tainan.TWN/11.09/5	N	N
				Mar 19				P	P
Cluster 5 (2009)									
** 34**	M	00/11	Mar 6	Mar 11	No	H1	MVs/Taipei.TWN/11.09/3	P	P
35	M	01/01	Mar 20	Mar 24	**Yes**	H1	MVi/Taipei.TWN/13.09	P	N
Cluster 6 (2009)									
36	M	19/09	Mar 27	Apr 15	*Yes*	G3	MVs/Pingtung.TWN/16.09/4	P	P
** 37**	M	21/11	Apr 7	Apr 9	*Yes*	G3	MVi/Kaohsiung.TWN/15.09/1	P	N
38	M	20/02	Apr 8	Apr 11	Unknown	G3	MVi/Kaohsiung.TWN/15.09/2	P	P
39	M	19/06	Apr 8	Apr 11	*Yes*	G3	MVs/Kaohsiung.TWN/15.09/3	P	P
40	M	23/04	Apr 9	Apr 11	*Yes*	G3	MVs/Kaohsiung.TWN/15.09/4	N	P
41	M	25/07	Apr 6	Apr 13	Unknown	G3	MVs/Kaohsiung.TWN/16.09/1	P	P
42	M	22/10	Apr 12	Apr 14	*Yes*	G3	MVs/Kaohsiung.TWN/16.09/3	N	P
43	M	21/00	Apr 12	Apr 15	Unknown	G3	MVs/Kaohsiung.TWN/16.09/5	P	P
44	M	21/04	Apr 14	Apr 20	Unknown	G3	MVi/Kaohsiung.TWN/17.09/2	P	N
45	F	00/05	Apr 20	Apr 22	No	G3	MVi/Kaohsiung.TWN/17.09/3	P	N
Cluster 7 (2009)									
** 46**	M	01/02	Apr 14	Apr 27	No	H1	–	P	P
47	M	38/08	Apr 26	May 12	Unknown	H1	MVs/Taichung.TWN/20.09	P	P
48	M	00/08	May 3	May 6	No	H1	MVi/Taichung.TWN/19.09/2	P	N
Cluster 8 (2009)									
** 49**	M	22/06	Apr 17	Apr 20	Unknown	H1	MVi/Taoyuan.TWN/17.09/1	P	P
50	M	33/00	Apr 30	Apr 30	*Yes*	H1	MVi/Taoyuan.TWN/19.09/1	N	P
Sporadic									
51	F	01/03	Feb 16	Feb 19	Yes	–	–	P	P
52	F	03/11	Mar 08	Mar 09	Yes	H1	MVi/Taichung.TWN/11.09/6	P	E
53	F	00/11	May 23	May 25	No	D8	MVs/Taipei.TWN/22.09	P	N

*The index case number for each cluster is denoted by boldface and underlining. – indicates PCR failed to obtain correspondence sequence for genetic analysis. Patients 16 and 29 provided samples twice. †The sampling year was 2009. ‡ Boldface indicates that these patients received the measles, mumps, rubella (MMR) vaccine within 2 weeks of rash onset. Italic indicates that the vaccination status was from patient or parent recall. §The strains were deposited into GenBank under accession numbers. GQ338677–GQ338680, HM072047–HM072085, and JF683608–JF683609 ¶ND, not done; N, negative; P, positive; and E, equivocal.
